# Vaginal Angiomyofibroblastoma: A Case Report and Review of Diagnostic Imaging

**DOI:** 10.1155/2018/7397121

**Published:** 2018-07-15

**Authors:** Sarah Eckhardt, Renee Rolston, Suzanne Palmer, Begum Ozel

**Affiliations:** ^1^Division of Female Pelvic Floor and Reconstructive Surgery, Department of Obstetrics and Gynecology, University of Southern California, Los Angeles, CA, USA; ^2^Clinical Radiology and Medicine, Keck Medical Center, University of Southern California, Los Angeles, CA, USA

## Abstract

**Background:**

Angiomyofibroblastoma (AMFB) is a benign mesenchymal tumor most commonly found in the female genital tract of premenopausal women. Although rare, AMFB is an important consideration in the differential diagnosis of vulvar and vaginal masses, as it must be distinguished from aggressive angiomyxoma (AA), a locally recurrent, invasive, and damaging tumor with similar clinical and pathologic findings.

**Case:**

We describe a patient with a 4 cm vaginal AMFB and the relevant preoperative radiographic imaging findings.

**Conclusion:**

Preoperative diagnosis of AMFB remains difficult. Common findings on magnetic resonance imaging and transvaginal sonography are described. We conclude that both transvaginal ultrasound and MRI are potentially useful imaging modalities in the preoperative assessment of vulvar and vaginal AMFB, with more data needed to determine superiority of one modality over the other.

## 1. Introduction 

Angiomyofibroblastoma (AMFB) is a rare, indolent mesenchymal tumor that most commonly occurs in the female genital tract in premenopausal women, most frequently in the vulva and vagina. AMFB typically measures less than 5 cm; however, case reports describe tumors of up to 23 cm in size [1]. Few cases have been reported affecting the fallopian tube, ischiorectal fossa, cervix, and bladder, as well as similar tumors in the male spermatic cord, scrotum, and perineum [2–6].

AMFB was first described in the literature by Fletcher et al. in 1992 as an important distinction from aggressive angiomyxoma (AA), which is an infiltrative myxedematous mesenchymal tumor with the potential for local recurrence [7].

Preoperative diagnosis of AMFB and distinction from other soft tissue tumors is often difficult, as there is limited information on characteristic imaging findings. The differential diagnosis for a vaginal or vulvar mass includes Bartholin's gland cyst, epidermal inclusion cyst, Gartner's duct cyst, fibroma, lipoma, hemangioma, leiomyoma, and alternative rare mesenchymal tumors. Here, we describe a case report of a vaginal AMFB and relevant radiologic features to aid in diagnosis and distinction from aggressive angiomyxoma.

## 2. Case

A 30-year-old gravida 1 para 1 female presented to our Emergency Department complaining of a vaginal mass present since the birth of her child 4 years earlier. At that time, she underwent an uncomplicated vacuum-assisted vaginal delivery and was unaware of significant lacerations or repairs. She felt that the mass had not changed significantly in size since the postpartum period, but she had never been evaluated by a physician. She complained of acutely worsening discharge over the previous month, described as watery yellow to pink and occasionally blood tinged. She denied changes in bowel movements, dysuria, hematuria, fevers, chills, night sweats, changes in appetite, or weight loss. She complained of both entry and deep dyspareunia.

**Figure 1:**
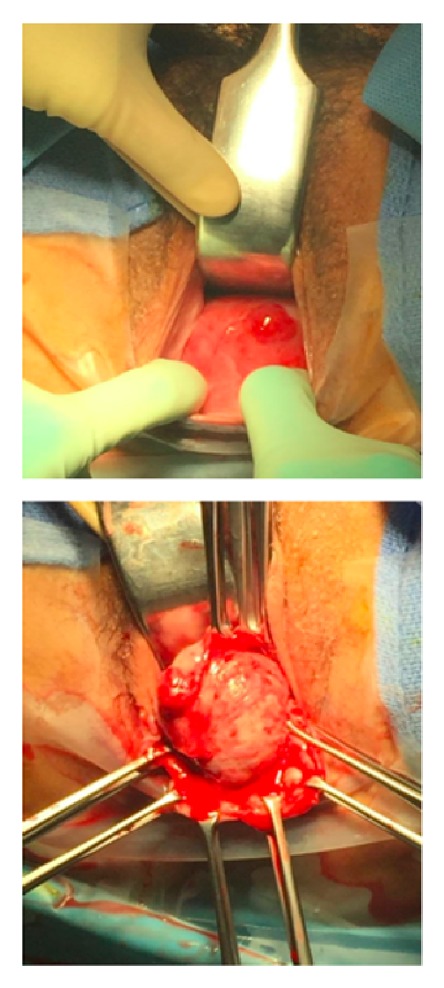
Lesion on exam under anesthesia and gross specimen during dissection.

Physical exam was notable for copious serosanguinous fluid within the vaginal vault. A well-circumscribed, smooth cystic structure approximately 4 cm in diameter was noted along the posterior vaginal wall. There was also a 0.5x1.0 cm exophytic lesion overlying the mass with serosanguinous drainage (Figure 1). On rectal exam, the mass was noted to be separate from the cervix and within the rectovaginal septum. Rectal involvement was not appreciated.

Tissue biopsies were taken; however, they were of limited diagnostic value, showing fibrous tissue with acute and chronic inflammation and squamous debris, consistent with cyst wall and contents.

**Figure 2:**
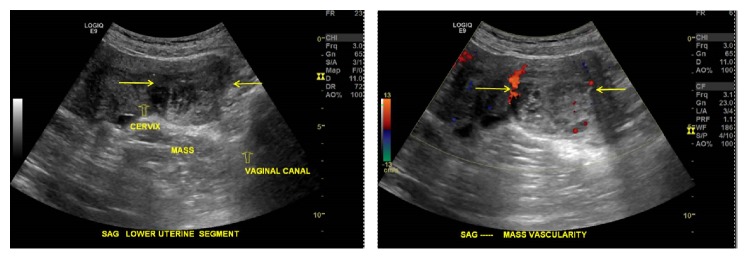
Transvaginal ultrasound. (a) Gray scale image shows a mixed echogenicity mass (arrows) with small hypoechoic cystic areas (3.0 MHz) 10 cm. (b) Color Doppler image shows minimal vascularity. Magnitude: 3 MHz.

**Figure 3:**
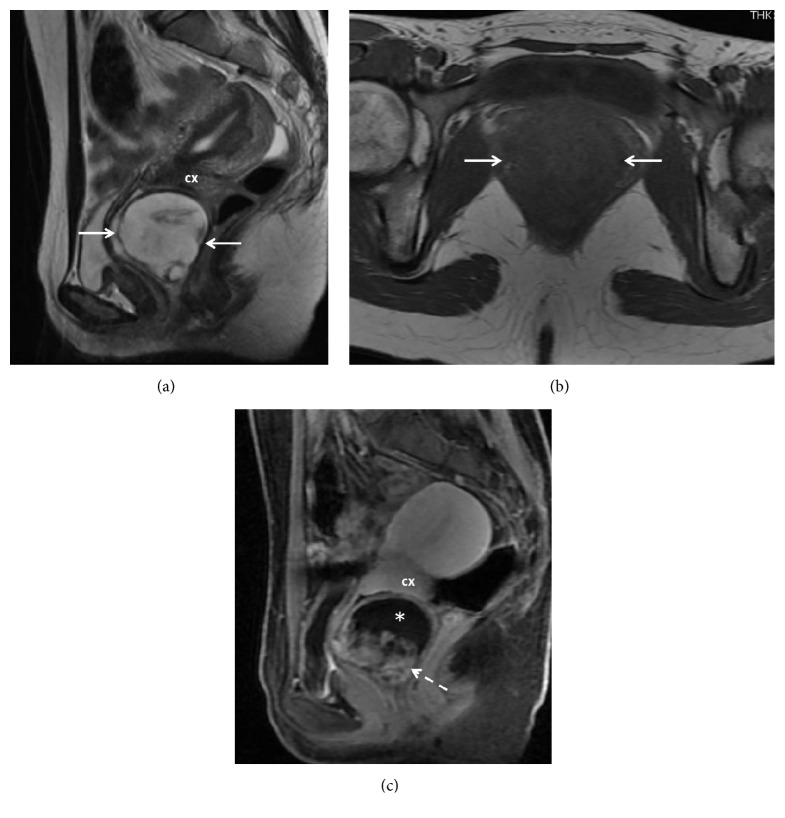
(a) Sagittal T2 weighted image shows a predominantly hyperintense mass (arrows) with small central areas of hypointensity. (b) On T1 weighted axial image the mass is homogeneously hypointense (arrows). (c) Sagittal contrast enhanced image demonstrates heterogeneous hyperenhancement inferiorly (dashed arrow) and absent enhancement superiorly (*∗*). Cervix: cx.

A pelvic ultrasound (US) was performed and showed a complex vaginal mass, inseparable from the cervix, measuring 5.1 x 3.8 x 5.4 cm (Figure 2(a)). Color Doppler demonstrated minimal peripheral vascularity (Figure 2(b)). Magnetic resonance imaging (MRI) was subsequently performed for further characterization (Figure 3). The mass measured 4.7 x4.8 x 4.9 cm and appeared to arise from the posterior wall of the vagina, separate from the cervix. The mass was heterogeneously hyperintense on T2-weighted (T2W) images and hypointense on T1-weighted (T1W) images. Postcontrast sequences demonstrated enhancement of the wall, absent internal enhancement superiorly, and bulky, nodular, hyperenhancement inferiorly, consistent with a complex cystic mass.

**Figure 4:**
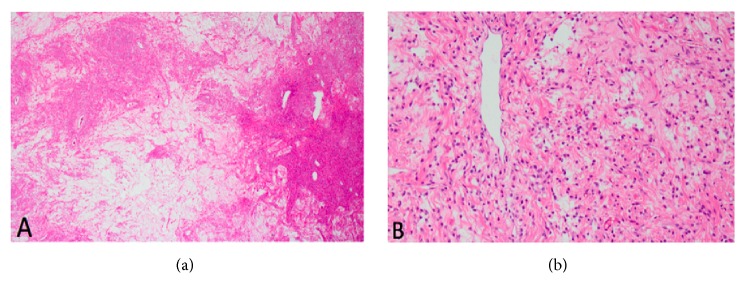
(a) Mesenchymal lesion with alternating hypercellular and hypocellular areas (4x magnification; hematoxylin and eosin stain). (b) Higher magnification demonstrating ovoid and spindle-shaped cells aggregated around small blood vessel (40x magnification).

The patient was taken to the operating room for an uncomplicated surgical excision. Histology was notable for hypocellular edematous myxoid stromal tissue alternating with hypercellular areas of stromal cells clustered around thin walled small to medium-sized vessels, consistent with angiomyofibroblastoma. Stromal cells were noted to be spindled with eosinophilic cytoplasm. Nuclei were round or ovular with fine chromatin (Figure 4). Rare mitotic figures were noted. Immunohistochemical stains were negative for desmin, alpha-smooth muscle actin (*α*-SMA), and progesterone receptor but demonstrated focal estrogen receptor positivity.

## 3. Comment

Although AMFB is a rare diagnosis, it is an important consideration in the premenopausal and perimenopausal patient presenting with a vulvovaginal mass given its predilection for this region of the female genital tract. In a review of the literature in 2015 by Wolf et al., 125 cases of female AMFB had been previously reported, of which 92% were either vulvar (N = 98) or vaginal in origin (N = 17). Median age was 45, and the majority of cases were observed in women less than 60 years of age [8]. Since 2015, there have been 10 additional cases including ours describing AMFB in females, six of which were located in the vulva, one cervical, one in the broad ligament, and one on the patient's foot [2, 9–17]. All women were under the age of 50.

Differential diagnosis for AMFB includes Gartner's duct cyst, epidermal inclusion cyst, leiomyoma, and fibroepithelial polyps among the more common etiologies. It is most important to distinguish AMFB from aggressive angiomyxoma (AA), which can commonly be misdiagnosed based on anatomic and pathologic similarities (Table 1). Although there have been no reports of distant metastasis, AA is known to recur in 33-72% of cases and is locally invasive, often entrapping nerves and mucosal glands [18, 19]. Surgical approach to AA requires wide local excision given the infiltrative nature of the lesion versus simple excision of AMFB; thus, it is important to attempt differentiation between the two prior to surgery.

Diagnostic utility of preoperative imaging for AMFB remains controversial. However, as the number of reported cases increases in the literature, more data exists on common radiographic features (Table 2).

### 3.1. Ultrasonography

US is typically the first imaging modality used to evaluate vaginal masses due to its intrinsic high resolution, availability, and cost effectiveness. US can be utilized to distinguish vulvar and vaginal AMFB from AA and other mesenchymal tumors [20, 21]. AMFB shows hyperechoic areas with irregular and small hypoechoic cystic areas interspersed within homogenous echogenic stroma [8, 20–23]. Minimal vascularity in AMFB is occasionally noted [8, 22]. Absence of prominent vascularity on color Doppler is consistent histologically with the predominant capillary-like vascular component of AMFB. Echogenic areas have been shown to represent hypocellular stroma on histology, while hypoechogenicity represents hypercellular areas [8, 21]. In our case, US characteristics were similar to those previously described.

In contrast, US appearance of AA is typically a hypoechoic mass with homogenous echogenicity and occasional echogenic septa correlating to fibrous bands on pathology [24, 25]. These fibrous bands can create a layered or swirling appearance similar to that seen on MRI and CT imaging of AA [26]. Vascularity can also be much more prominent on color Doppler of AA than AMFB [26, 27].

To complete the differential diagnosis, vaginal wall cysts are anechoic masses without internal vascularity; vaginal epidermoid cysts are homogeneous hyperechoic masses without internal vascularity; and heterogeneous solid masses are often malignancies or leiomyoma [22].

### 3.2. Magnetic Resonance Imaging

On MRI, AMFB is typically well circumscribed [9]. It is rare to see central necrosis or degeneration but it does not rule out AMFB [9]. AMFB is most often described as hypointense on T1 weighted (T1W) images, similar to that of skeletal muscle, hyperintense on T2 weighted (T2W) images, and with homogenous hyperenhancement on gadolinium chelate (Gd-C) enhanced images [1, 9, 23, 28]. Differences in T1W and T2W signal intensity may be attributable to variations in lipid and collagenous content in AMFB [8, 23, 28–31].

Contrast enhanced imaging was only reported in 5 studies. Four showed homogenous hyperenhancement and one demonstrated heterogeneous enhancement [23]. Hyperenhancement is thought to be related to prominent vascularity associated with myofibroblast tumors and AMFB in particular [1, 9, 23, 28, 30–32].

Our case demonstrated heterogeneously high T2 and low T1 signaling intensity on MRI which is consistent with the literature. Postcontrast sequences demonstrated enhancement of the wall, absent internal enhancement superiorly, and bulky nodular enhancement inferiorly, consistent with a complex cystic mass.

When correlated histologically, hyperintensity on T2W and absent enhancement correspond to hypocellular areas with abundant collagenous stroma. Areas of hyperenhancement correspond to areas of hypercellularity and vascularity, with little collagenous stroma and water content. Intermediate intensity on T2W and less avid enhancement are thought to represent areas of intermediate cellularity and collagenous stroma [28].

Data on MRI characteristics of AA is limited. AA has similar findings to AMFB on T1W and T2W images but is thought to be differentiated best by its enhancement characteristics. AA is described as having an intense, swirled or layered pattern. Similar swirling and layered enhancement is also described on contrast enhanced CT [26–28, 33–36]. AMFB does not typically show infiltrative pattern but it can grow around structures. If infiltration and swirled or layered pattern are observed, AA is much more likely than AMFB [28, 34–36].

## 4. Conclusion

In conclusion, in the patient with a vaginal or vulvar mass, it is important to consider AMFB among the differential diagnoses, particularly because of the importance of distinguishing AMFB from AA for treatment purposes. Postoperative histologic examination is needed for definitive diagnosis; however, we propose that ultrasound and MRI are increasingly useful diagnostic tools, particularly as the number of reported cases increases. Given the cost effectiveness of ultrasound, initiating imaging workup of a solid vaginal or vulvar mass with ultrasonography is suggested.

## Figures and Tables

**Table 1:** Clinical and pathologic features of AMFB and AA.

	**AMFB**	**AA**
Clinical features		
** Age (years)**	16 – 86 (Median 45)	16 – 70 (Median 37)
** Size (cm)**	< 5 (0.5 – 23)	>5 (1-60)
** Location**	Female pelvis and perineum: predominantly vulvar/vaginal	Female pelvis and perineum
** Onset**	1 month to 8 years	1 month – 5 years
	Predominantly 1-2 years	Predominantly < 1 year
** Recurrence**	None reported	33-72%
Pathology		
** Low-power features**	Well-defined	Infiltrative
	No entrapment of mucosal glands or nerve bundles	Entrapped mucosal glands and nerve bundles
	Alternating hypocellular and hypercellular areas	Stromal cells distributed throughout
** Vasculature**	Abundant thin-walled vessels, mostly capillary-like	Small to medium-sized vessels, mostly thick-walled or hyalinized
** Stromal cells **	Abundant	Low cellularity
	Spindle, plump spindle, or oval	Thin delicate spindle or stellate
	Perivascular aggregation	Mitotic figures typically absent
	Mitotic figures typically absent	
** Stroma**	Mucin-poor, containing delicate, wavy collagen fiber	Myxoid, hyaluronic-acid rich
Immunostaining		
** Desmin**	Positive (50-60%)	Positive (Up to 73%)
** ** **α** **–SMA**	Negative (positive in up to 15%)	Positive
** S-100**	Negative	Negative
** Vimentin**	Positive	Positive
** Estrogen receptor**	Positive	Positive
** Progesterone receptor**	Positive	Positive

**Table 2:** Characteristic imaging features of AMFB on MRI and US.

**Study**	**Sex**	**Age**	**Size (cm)**	**Location**	**MRI**	**TVUS**
**Wolf et al. **	F	38	9x6x1.5	Vagina	T1 weighted: Homogenous hypointense	Well-demarcated, homogenous mass, medium echogenicity, few septations
					T2-weighted: Homogenous hypointense mass	Doppler: several small intralesional vessels

**Souza et al.**	M	19	2.8	Scrotum	T1-weighted: Central hypointensity Intermediate hyperintensity, capsule	Well-defined, heterogeneous echo texture, small hypoechoic cysts
					T2-weighted: Intermediate homogenous hyperintensity, Hypointense capsule Central heterogeneous mixed hypo and hyperintensity	Doppler: minimal flow

**Shoji et al.**	F	50	8x7x5	Vulva	T1-weighted: Hypointense mass	N/A
					GA-T1: Homogenous enhancement, poorly enhanced area at center	
					T2-weighted: Mildly hyperintense	

**Geng et al. **	F	46	8.5x6x16	Paravaginal	T1-weighted: signal intensity similar to skeletal muscle	N/A
					GA-T1: Homogenous hyperintensity	
					T2-weighted: Hyperintensity, mild hyperintensity mild hypointensity	

**Kim et al.**	F	40	9x5.5x2.5	Vulva	N/A	Mixed echoic soft tissue massHyperechoic areas mixed with irregular hypoechoic areas with tiny hypoechoic components
						Doppler: negative

**Qiu et al.**	F	32	13.2x5.8 x7.8	Posterior cul-de-sac	N/A	Moderately echoic mass, small hypoechoic area
						Doppler: N/A

**Kitamura et al.**	F	24	3 x 2.8 x 2.8	Urethra	T1-weighted: N/A	N/A
					T2 Weighted: homogenous, well-defined, low to moderate hyperintensity	

**Lim et al. **	F	48	3.8x3.5x2.8	Posterior perivesical space	T1-weighted: Signal intensity similar to skeletal muscle	N/A
					GA-T1: Strong homogenous enhancement	
					T2-weighted: well-defined mass heterogeneous intermediate signal intensity, focal nodular and central curvilinear dark signal intensities	

**Maruyama et al. **	M	72	7.2x5.5x2.2	Scrotum	T1-weighted: Similar to or lower signal intensity than skeletal muscle	Mixed echogenicity, with several small hypoechoic cystic areas^*∗*^
					GA-T1: strong heterogeneous enhancement	
					T2-weighted: heterogeneous intermediate to high signal intensity	

**Wang et al.**	F	N/A	N/A	Anterior vaginal	N/A	Poorly defined, irregularly shaped, hypoechoic, band-like echoes of various width
						Doppler: positive blood flow

**Wang et al.**	F	N/A	N/A	Urethral	N/A	Well-defined, hypoechoic cystic solid mass
						Doppler: positive blood flow

**Mortele et al.**	F	46	Perineal	2.5x3.5	T1-weighted: hypointense, inhomogeneous mass with small focus of hyperintensity representing fat	N/A
					GA-T1: Strong homogenous uptake	
					T2-weighted: Hyperintense homogenous signal similar to surrounding fat	

## Data Availability

The magnetic resonance imaging and ultrasound data used to support the findings of this study can be found within the cited articles in this study. Please refer to Table 2 for a complete list of imaging studies included in this review. Patient images used to support findings of this case report study are available from the corresponding author upon request.

## Additional Points

*Precis*. Ultrasound and magnetic resonance imaging are useful modalities in the diagnosis of angiomyofibroblastoma, a rare mesenchymal tumor. Teaching Points. (1) AMFB occurs in the vulva and vagina in over 90% of reported cases: AMFB should be considered on the differential diagnosis of benign appearing vulvar and vaginal masses. (2)Transvaginal ultrasound is the recommended first line imaging for AMFB. MRI is recommended as second-line imaging and is preferable to CT. (3) It is important to attempt preoperative distinction from aggressive angiomyxoma in order to optimize surgical approach.
